# Predictive analysis of ratings of perceived exertion in elite Gaelic football

**DOI:** 10.5114/biolsport.2024.134753

**Published:** 2024-03-06

**Authors:** Dermot Sheridan, Aidan J. Brady, Dongyun Nie, Mark Roantree

**Affiliations:** 1 School of Computing, Dublin City University, Dublin, Ireland; 2 Insight Centre for Data Analytics, Dublin City University, Dublin, Ireland

**Keywords:** Athlete monitoring, External load, Global positioning systems, Machine learning, Team sports

## Abstract

This study aimed to compare the predictive accuracy of absolute and relative external load indices (ELI) across three machine learning models, and predict the rating of perceived exertion (RPE) of elite Gaelic football players using ELI, personal characteristics, wellness scores, and training workloads. ELI and related variables were collected from 49 elite Gaelic football players over three competitive seasons resulting in 1617 observations. ELI included total distance, high speed running distance (≥ 4.72 m · s^−1^), and number of accelerations and decelerations (n ± 3 m · s^−2^), expressed in both absolute and relative terms. Variables related to personal characteristics, wellness scores, and training workloads were also included. Data were analysed using decision tree, random forest (RF), and bootstrap aggregation (BS) models. The RF model had the highest predictive accuracy using absolute and relative ELI only, at 54.3% and 48.3%, respectively. Total and relative distance were the strongest predictors of RPE in the RF model, accounting for 38.8% and 27.9% of the normalised importance. The BS model had the highest accuracy at 67.0% and 65.2% for absolute and relative ELI when performed in conjunction with the related variables, respectively. The current models demonstrate potential to predict RPE and subsequently optimise training load in Gaelic football.

## INTRODUCTION

Effective monitoring and manipulation of an athletes training load is central to eliciting improvements in physical performance, and limiting the risk of injury and/or illness [1]. In invasion field-based team sports (IFTS) such as soccer, Gaelic football, and rugby, training load is typically quantified using external, internal, or a combination of external and internal load indices [2, 3]. External load represents the physical work performed by a player during training or match-play [4]. Internal load represents the psycho-physiological response incurred in response to an external stimulus during training or match-play [1]. Global positioning system technology (GPS) provides large quantities of valid and reliable data pertaining to a players speed and distance that can be quantified in real-time and is the most common method of external load monitoring in IFTS [5, 6]. Internal load can be quantified objectively using blood lactate and/or heart rate, or subjectively using ratings of perceived exertion (RPE). In IFTS, a RPE-based approach is widely used as it is valid, non-invasive, and low cost [7, 8].

The collective nature of IFTS training and use of drill based scenarios such as small sided games [9] means players are regularly prescribed the same external load. Training prescription based on external load indices (ELI) can however, result in considerable inter-individual variation in RPE [10–12]. This is an important consideration when manipulating external load as it is the internal load that promotes adaptation [13]. Prescription of training using internal load indices such as RPE are therefore desirable. However, RPE can only be collected following the cessation of training or match-play, with large differences between coaches intended RPE and athletes subsequent RPE [14, 15].

A limited number of studies have attempted to predict RPE using machine learning models by incorporating a combination of ELI and related contextual factors such as the athletes physical fitness level, personal characteristics, wellness scores, and training and match history [11, 16–19]. Using artificial neural networks (ANN) RPE was more accurately predicted compared to traditional statistical approaches using total distance, relative distance, absolute high speed running (> 4.0 m · s^−1^) and percentage of total distance at high speed [root mean square error (RMSE) 1.85 vs. 1.42] for a group of Australian football players [16]. Total distance was identified as having the highest importance score for predictive accuracy [16]. More recently, using eight relative ELI collected during soccer training in a decision tree (DT) model, RPE prediction had a RMSE of 1.62 with relative high speed running (> 5.5 m · s^−1^) having the highest importance score [18]. Inclusion of heart rate percentages, and variables pertaining to the athletes individual characteristics improved the predictive accuracy of a gradient boosting machine in soccer with a RMSE of 0.93 although only 47.6% of predictions were correct [17].

While these models demonstrate value for predicting RPE, lack of consistency in the variables incorporated in the models, in particular the lack of standardisation of the ELI, and differences in machine learning models between studies limit the generalisability of these results to other IFTS such as Gaelic football. Further, although soccer, Australian football, rugby and Gaelic football are IFTS, players in each sport have a unique activity profile due to several factors including differences in playing rules, pitch size and playing time [20]. For example, the relative playing area per player is ~425 m^2^ in Gaelic football, compared to ~320 m^2^ in soccer [21]. Further analyses are therefore required to examine differences between IFTS and which indices have the greatest influence on model accuracy.

The aims of this study were to compare the accuracy of absolute and relative ELI across DT, random forest (RF), and bootstrap aggregation (BS) models in predicting RPE for a cohort of elite Gaelic football players and to examine the predictive accuracy of these models using ELI, personal characteristics, wellness scores, and training workloads. It was hypothesised that accuracy would be higher using absolute ELI, and that inclusion of variables related to personal characteristics, wellness scores, and training workloads would improve the accuracy of the DT, RF, and BS models.

## MATERIALS AND METHODS

### Participants

Forty-nine elite Gaelic football players (mean ± standard deviation (SD), age, 25.6 ± 4.0 y; height, 1.82 ± 0.06 m; body mass, 82.0 ± 7.1 kg) from one inter-county team gave written informed consent to participate in this study. The team were competing in Division 2 or Division 3 of the National Football League during the data collection period. Inclusion criteria was limited to outfield players. Ethical approval was obtained from Dublin City University Research Ethics Committee (DCU/REC/2021/267) in accordance with the Declaration of Helsinki.

### Experimental design

GPS technology was used to measure the activity levels of elite Gaelic football players during training and match-play in the 2020, 2021 and 2022 inter-county seasons. RPE was recorded from players on a one-to-one basis following the completion of each training session and match. Training sessions were limited to field-based sessions, and were completed on a grass playing surface of approximately 140 m in length and 80 m in width. A total of 1616 GPS data files and concomitant measures of RPE were recorded. This included 96 training sessions and 44 matches resulting in 1205 and 411 records, respectively. A total of 562, 575, and 479 records were collected during the 2020, 2021, and 2022 playing seasons. The median (range) number of observations per player was 29 (2–98) with a mean ± SD of 32.6 ± 22.9.

### Data collection

RPE was measured using the modified Borg CR10 scale as outlined previously [8]. Ratings were recorded approximately 30 min after each training session and match using a mobile application (Smartabase, v.6.8.08, Fusion Sport, Milton, Australia). The use of a mobile application allowed ratings to be recorded privately, without the influence of peer presence or other related environmental factors [22]. All players were familiar with the use of the CR10 scale prior to participation in the present study. RPE was subsequently categorised as low (RPE ≤ 5, n = 461), moderate (RPE 6–7, n = 710), and high (RPE ≥ 8, n = 446). These categories have been used previously in IFTS [12, 23] and are associated with the three physiological exercise intensity domains [24].

External indices of activity were collected using GPS units sampling at a rate of 18 Hz (GPEXE LT, Exelio, Italy). During each training session and match, players wore an individual GPS unit that was positioned between the scapulae in a custom-made undergarment. These units have shown *good* to *moderate* (< 10% typical error of estimate) validity, and *good* (< 5% coefficient of variation) reliability for distance covered across a range of movement speeds in a team-sport specific circuit [5, 25]. Following each training session and match, data was downloaded to the manufacturers proprietary software (GPEXE Bridge v.6.9.25) to remove values unrelated to the training session or match. Forty-two distinct movement variables related to speed and distance were extracted from each GPS data file. Prior to inclusion in the models, multicollinearity tests were performed for all GPS variables. Where the variance inflation factor (VIF) exceeded 5, variables were sequentially removed in line with expert knowledge on the practical utility of each [2]. The remaining movement variables included in the models were total distance, high speed distance (≥ 4.72 m · s^−1^), and number of accelerations and decelerations (n ± 3 m · s^−2^) expressed both in absolute terms independent of playing time, and relative terms in metres and distance per unit time.

Stature was measured to the nearest 0.1 cm using a portable stadiometer (model 213, SECA, Hamburg, Germany). Body mass was measured to the nearest 0.1 kg using a portable digital scale (model 813, SECA < Hamburg, Germany). Lean mass index and body fat percentage were assessed using duel-energy x-ray absorptiometry (DEXA). Age, body mass index, playing position, and playing experience, classified as the number of years playing at the elite level of Gaelic football, were also included. Maximal aerobic speed (MAS) was determined using the time taken to complete a 1200 m time-trial (TT). The TT was performed at the beginning of each playing season. Where a player was unable to complete the TT (4%), the group mean was entered as their MAS.

Perceived wellness was examined prior to each training session and match using a 5 item questionnaire that was submitted through a mobile application (Smartabase, v.6.8.08, Fusion Sport, Milton, Australia). Each item was rated on a 10-point scale. The individual items were subsequently categorised as muscle soreness, sleep quality, or energy levels using the average score of each item in the section. To provide greater information on the data and time of each training session or match, session time (am or pm), day of the week, month, and season were included in the models. Activity type and days to and from the next and previous training session or match, respectively, were also included. Acute:chronic workload ratio (ACWR) indices were used to monitor changes in workload over a given training period. A total of 21 indices were generated. After examination of multicollinearity, four ACWR variables remained which were 7-d total distance, 28-d total distance, ACWR total distance, and ACWR high speed distance.

### Statistical analysis

To examine the performance of ELI, with and without the inclusion of related variables, in the prediction of RPE, three machine learning models were performed on four occasions each. The machine learning models were DT, which create a tree-like graph of decisions based on the values of features, RF, which creates an ensemble of DT where a combination of learning models increases the overall result, and BS, which is another ensemble method that combines the predictions from multiple machine learning algorithms to make more accurate predictions. The first two iterations of each model were used to examine the difference in predictive accuracy between the absolute and relative ELI, independent of related variables. The third and fourth iterations examined the predictive accuracy of the absolute and relative external load variables together with perceived wellness, ACWR, and athlete-specific variables, respectively.

The variable sets included in the third and fourth iteration of each model were examined for strength of association prior to inclusion in the models using the Spearman rank-order correlation coefficient. The magnitude of association was interpreted as trivial (0.0 to 0.1), small (0.1 to 0.3), moderate (0.3 to 0.5), large (0.5 to 0.7), very large (0.7 to 0.9), or almost perfect (0.9 to 1.0) [26]. Where the correlation coefficient exceeded 0.5, variables were removed in line with expert consensus on the practical importance of each. All variables were subsequently normalised prior to inclusion in the models. The categorisation of RPE resulted in an unbalanced number of records in each category. A down-sampling method was therefore employed to take the smallest number of records from each category and randomly select an equal number of records for the other RPE categories (n = 446).

The DT, RF, and BS models require a learning dataset to construct the model, and a testing set to evaluate the models performance. The learning set in the present study consisted of 80% of the data while the testing set contained the remaining 20%. Importance scores for each variable in making correct predictions were calculated from the DT and RF models. The importance score of each variable is a measure of the magnitude by which the model-predicted RPE differs between the values of each variable. The importance score was determined through the number of expressions of a variable in the created DT. All models were performed in Python (v.3.5) programming software (Python Software Foundation, Wilmington, DE, USA). A one-way analysis of variance was also performed to examine the differences in each model variable across the low, moderate, and high RPE categories. When a significant effect of group was indicated, *post hoc* testing was then performed with Bonferroni correction. Data are presented as mean ± SD, unless otherwise stated. The significance level was set at α ≤ 0.05 for all tests.

## RESULTS

**FIG. 1:**
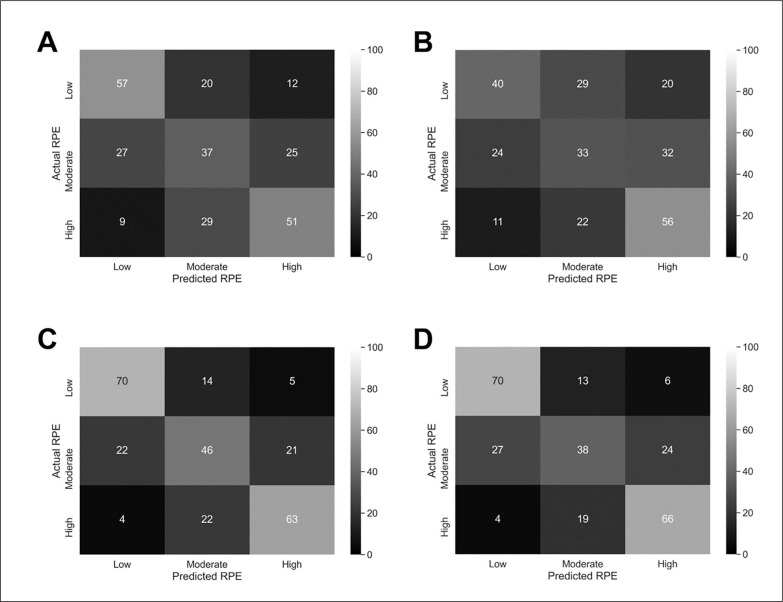
Confusion matrix of the actual RPE and predicted RPE for the (A) RF model using absolute ELI only, (B) RF model using relative ELI only, (C) BS model using absolute ELI and related variables, and (D) BS model using relative ELI and related variables.

The mean ± SD of each model variable in the low, moderate, and high RPE categories is presented in Table 1. The accuracy of the DT, RF and BS models for predicting RPE for each dataset is presented in Table 2. The RF model had the highest accuracy score at 54.3% and 48.3% when using absolute and relative ELI, respectively. The normalised importance score for each absolute and relative external load measure in the RF models is presented in Table 3 and Table 4, respectively. For absolute ELI, total distance had the highest importance score at 0.388. Relative distance had the highest importance score of the relative ELI at 0.279. The BS model had the highest predictive accuracy after inclusion of variables related to personal characteristics, wellness scores, and training workloads alongside the absolute and relative ELI at 67.0% and 65.2% accuracy, respectively. The accuracy of the RF and BS models for the low, moderate and high RPE categories are presented in a confusion matrix in Figure 1.

**TABLE 1:** Descriptive analysis of each model variable

Variable	Low (RPE ≤4)	Moderate (RPE 5 – 7)	High (RPE ≥8)	P value
Age (yr)	26.1 ± 4.1	25.7 ± 4.2	24.4 ± 3.7^c^	0.018
Lean mass index (m · kg^2^)	21.0 ± 1.4	20.7 ± 2.3	20.6 ± 1.3	0.333
Body fat percentage (%)	14.3 ± 4.0	14.8 ± 3.6	14.2 ± 4.0	0.484
Maximal aerobic speed (m · s^−1^)	4.73 ± 0.20	4.75 ± 0.20	4.72 ± 0.24	0.595
Duration (min)	60.6 ± 21.0	72.1 ± 22.8^b^	83.5 ± 27.0^a,e^	< 0.001
Total distance (m)	4951 ± 1457	6128 ± 2000^b^	8140 ± 3131^a,d^	< 0.001
Relative distance (m · min^−1^)	84.3 ± 18.4	88.1 ± 22.0	98.7 ± 21.3^a,e^	< 0.001
High speed distance (≥4.72 m · s^−1^)	816 ± 478	921 ± 537	1234 ± 675^a,d^	< 0.001
Relative high speed distance (≥4.72 m · s^−1^)	14.1 ± 7.7	13.9 ± 8.6	15.3 ± 8.1	0.450
Accelerations (n)	9.8 ± 6.7	12.2 ± 8.3	12.1 ± 7.3	0.053
Relative accelerations (n · min^−1^)	0.2 ± 0.2	0.2 ± 0.2	0.2 ± 0.1	0.490
Decelerations (n)	6.2 ± 4.8	9.4 ± 6.1^b^	14.1 ± 8.0^a,d^	< 0.001
Relative decelerations (n · min^−1^)	0.1 ± 0.1	0.1 ± 0.1	0.2 ± 0.1^a^	< 0.001
Muscle soreness (AU)	5.0 ± 2.7	5.4 ± 2.8	5.3 ± 2.7	0.651
Sleep quality (AU)	7.9 ± 1.3	8.2 ± 1.0	8.0 ± 1.0	0.082
Energy level (AU)	7.8 ± 1.2	7.9 ± 1.0	7.7 ± 1.5	0.633
7-d total distance	14031 ± 6880	15958 ± 5889	17115 ± 6882^b^	0.007
28-d total distance	47846 ± 23520	44207 ± 21012	38291 ± 22223^c^	0.016
ACWR total distance	0.95 ± 0.19	1.03 ± 0.17^b^	1.05 ± 0.15^a^	< 0.001
ACWR high speed distance	1.03 ± 0.33	1.03 ± 0.37	1.08 ± 0.25	0.463
Days to last match	5.2 ± 5.4	3.5 ± 4.7^c^	1.1 ± 3.2^a,d^	< 0.001
Days to next match	4.1 ± 3.4	7.1 ± 5.5^a^	9.4 ± 5.7^a,e^	< 0.001

ACWR, acute:chronic workload ratio.

a , P < 0.001 vs. Low;

b , P < 0.01 vs. Low;

c , P < 0.05 vs. Low;

d , P < 0.001 vs. Moderate;

e , P < 0.01 vs. Moderate.

**TABLE 2:** Accuracy of the decision tree, random forest, and bootstrap aggregation models for each dataset

	Decision tree	Random forest	Bootstrap aggregation
Absolute ELI	48.7%	54.3%	52.8%
Relative ELI	47.6%	48.3%	42.3%
Absolute ELI and related variables	57.7%	61.0%	67.0%
Relative ELI and related variables	58.4%	61.0%	65.2%

ELI, external load indices.

**TABLE 3:** Normalised importance scores from the random forest model for absolute external load indices.

Variable	Importance score
Total distance (m)	0.388
High speed distance (≥ 4.72 m · s^−1^)	0.259
Decelerations (n)	0.193
Accelerations (n)	0.160

**TABLE 4:** Normalised importance scores from the random forest model for relative external load indices.

Variable	Importance score
Relative distance (m · min^−1^)	0.279
Relative accelerations (n · min^−1^)	0.256
Relative decelerations (n · min^−1^)	0.249
Relative high speed distance (≥ 4.72 m · s^−1^)	0.216

## DISCUSSION

The aims of the present study were to compare the accuracy of absolute and relative ELI across three machine learning models when predicting RPE, examine the predictive accuracy of the three models using ELI alongside variables related to personal characteristics, wellness scores, and training workloads, and compare the accuracy between RPE categories. The RF model had the highest accuracy for predicting RPE at 54.3% and 48.3% using only absolute and relative ELI, respectively. Inclusion of related variables alongside the ELI improved the predictive accuracy of the three models, which was highest using the BS model at 67.0% and 65.2% for the absolute and relative datasets, respectively.

Previous studies that have attempted to predict RPE from external load have used absolute [17], relative [18], or a combination of both absolute and relative ELI [11] yet none have compared the predictive accuracy between absolute and relative indices. Comparison of these indices will enable a more objective approach to variable selection in future predictive models. A DT, RF, and BS model were run on absolute ELI, followed by relative ELI resulting in six different results sets for comparison. In all models, ELI expressed in absolute terms had a greater accuracy by 1.1–10.5%. The RF model, which had the best accuracy for both absolute and relative ELI, was 6% higher using the absolute indices. A recent examination of the contribution of training intensity and duration to training load in rugby league and rugby union reported that session duration accounted for a larger proportion of the total variance in training load than session intensity [27]. This supports the findings of the present study in that ELI, expressed in absolute terms, appear to be more influential on RPE although relative ELI are more commonly reported in IFTS [6].

In the present study, RPE was divided into three categories. This approach has been used previously when examining factors influencing RPE in IFTS [12], and with other cohorts, including endurance athletes [28]. The boundaries of these categories are reflective of the first and second ventilatory thresholds [24], and may be used to demarcate entry into the three distinct physiological exercise intensity domains [29]. This approach may be more practically relevant given the differing physiological adaptations that can occur across the exercise intensity domains, and provide coaches with a larger target window when using RPE for training prescription [23].

The RF model in the present study correctly predicted 64%, 42% and 57% of cases in the low, moderate and high RPE categories using absolute ELI. When using relative ELI, the predictive accuracy in the low, moderate, and high RPE categories was 45%, 37%, and 63%, respectively. The width of the moderate category may have contributed to the lower predictive accuracy compared to the low and high RPE categories. It is difficult to compare the results of the present study with previous research as most other models have attempted to predict the specific RPE value rather than a distinct class [11, 17, 18]. However, one study reported an accuracy of 47.6% for single RPE values which was increased to 91.7% when using a ‘loose accuracy’ approach, where predictions within a range of ± 1 unit of the actual value were marked correct [17] demonstrating the similarities of predictions at the boundary values. Notably, the latter study used a combination of ELI and a range of related variables yet had a lower accuracy score than both RF models in the present study using ELI only.

The accuracy score across RPE categories was lowest in the moderate category. This is likely due to the boundary overlap with the low and high RPE categories and the moderate category having the narrowest range. A surprising finding of the present study was that accuracy in the high category considerably outperformed the low category in the relative model. It is plausible that the relative model performed better in the high category as the differences between the ELI were more pronounced. For example, relative distance differed by 3.8 m · min^−1^ between the low and moderate categories but differed by 10.6 m · min^−1^ between the moderate and high category. By contrast, the low category outperformed the high category in the absolute model, although the difference was less pronounced.

Machine learning allows for the extraction of importance scores for the variables included in the RF models. As variable importance is calculated on the number of variables used in the model, there is a direct effect on the calculation of importance with the complexity of the algorithm and the number of variables considered. An understanding of the contribution of each variable to the predictive accuracy of RPE can allow more effective planning and control of training loads. In the present study, total distance had the highest importance score of the four absolute and the four relative ELI. This is in agreement with a recent meta-analysis where total distance had the strongest association with RPE [3] and the abovementioned analysis in rugby league and rugby union [27]. Equally, total distance was the strongest predictor of RPE using ANN [16]. By contrast, relative high speed running (> 5.5 m · s^−1^) was the strongest predictor of RPE, accounting for 61%, with relative distance having the lowest score in youth soccer when using a DT model [18]. These differences may be due, at least in part, to differences in the activity profile of Gaelic football and soccer, and the role of player age as youth players report higher RPE values in training than adult players [30, 31]. The type of machine learning model and high speed running thresholds may also have contributed to these differences.

The inclusion of a range of variables alongside ELI provided six additional results sets. This improved the predictive accuracy of all three models in the present study. Of note, the BS model improved by 14.2% and 12.9% using variables related to personal characteristics, wellness scores, and training workloads alongside the absolute and relative ELI, respectively. The BS model outperformed both the DT and RF models after inclusion of related variables although the DT and RF models improved by an average of 9.9% and 9.7%, respectively. The inclusion of a wide variety of variables related to athletic performance has been recommended to provide a more holistic approach [17, 19]. Those chosen for inclusion in the present study were factors shown to influence RPE and the activity performed during Gaelic football, such as body composition, player experience, physical fitness levels, accumulated training load, and sleep quality and muscle soreness [32–34], and those which have improved the predictive accuracy of previous models, such as individual characteristics and supplementary variables that contributed 4.5–33% accuracy in soccer [17]. The accuracy in the low RPE category improved to 78.7% in both BS models. In the high RPE category, accuracy improved to 70.8% and 74.2% alongside the absolute and relative ELI, respectively. These findings demonstrate the importance of including variables beyond ELI to predict RPE and highlight the potential practical application to IFTS.

Unlike the RF models, the contribution of each variable in the BS models cannot be quantified making it difficult to discern which factors are of greatest importance and should be prioritised during training and match-play, particularly those which were significantly different between groups such as days to/from last match and cumulative distance. However, the variables included in the present study can be collected in a relatively low cost, time-efficient manner and are already routine practice within many IFTS [1]. The relatively small number of training observations is a limitation of the present study. A larger dataset in machine learning can potentially improve the accuracy of the model by providing more representative data, reducing variance, and improving feature representation, but the quality and relevance of the data are also important factors that must be considered. The data collection was limited to a single team over a three-season period which occurred during a global pandemic that disrupted the normal training schedule and may have influenced the fitness levels of participants due to restricted movements and limited collective training.

### Practical applications

The findings of the present study highlight that for both absolute and relative ELI, distance covered has the greatest contribution to the predictive accuracy while the contribution of high speed running, accelerations and decelerations is largely similar. Coaches and practitioners should however, be cognisant of the differences in predictive accuracy between absolute and relative ELI and the subsequent changes in the importance score of each variable, particularly when using these variables to prescribe training and develop training programmes. The improved accuracy scores following inclusion of variables related to personal characteristics, wellness scores, and training workloads demonstrates that ELI alone do not adequately capture or predict RPE. A more holistic approach is therefore recommended. The higher accuracy scores in the low and high RPE categories compared to the moderate category suggests that when using RPE to predict a players response to training, a polarised training approach consisting of low volume, high intensity efforts and high volume, low intensity efforts may be the most suitable approach and increase the likelihood of eliciting the desired physiological stimulus.

## CONCLUSIONS

The accuracy of the three machine learning models to predict RPE in a cohort of elite Gaelic football players was higher when using absolute ELI compared to relative ELI emphasising the importance of volume over intensity. The inclusion of variables related to personal characteristics, wellness scores, and training workloads increased the accuracy of all three models, in particular a BS model which performed strongly in the low and high RPE categories. These models may be used to assist coaches and practitioners in planning, monitoring, and evaluating the demands of training and match-play.

## References

[1] Bourdon PC, Cardinale M, Murray A, Gastin P, Kellmann M, Varley MC, Gabbett TJ, Coutts AJ, Burgess DJ, Gregson W, Cable NT. Monitoring Athlete Training Loads: Consensus Statement. Int J Sports Physiol Perform. 2017; 12(s2):161–70. 28463642 10.1123/IJSPP.2017-0208

[2] Akenhead R, Nassis GP. Training Load and Player Monitoring in High-Level Football: Current Practice and Perceptions. Int J Sports Physiol Perform. 2016; 11(5):587–93. 26456711 10.1123/ijspp.2015-0331

[3] McLaren SJ, Macpherson TW, Coutts AJ, Hurst C, Spears IR, Weston M. The Relationships Between Internal and External Measures of Training Load and Intensity in Team Sports: A Meta-Analysis. Sports Med. 2018; 48(3):641–58. 29288436 10.1007/s40279-017-0830-z

[4] Impellizzeri FM, Marcora SM, Coutts AJ. Internal and External Training Load: 15 Years On. Int J Sports Physiol Perform. 2019; 14(2):270–3. 30614348 10.1123/ijspp.2018-0935

[5] Scott MTU, Scott TJ, Kelly VG. The Validity And Reliability Of Global Positioning Systems In Team Sport: A Brief Review. J Strength Cond Res. 2016; 30(5):1470–90. 26439776 10.1519/JSC.0000000000001221

[6] Whitehead S, Till K, Weaving D, Jones B. The Use of Microtechnology to Quantify the Peak Match Demands of the Football Codes: A Systematic Review. Sports Med. 2018; 48(11):2549–75. 30088218 10.1007/s40279-018-0965-6 PMC6182461

[7] Coutts AJ, Rampinini E, Marcora SM, Castagna C, Impellizzeri FM. Heart rate and blood lactate correlates of perceived exertion during small-sided soccer games. J Sci Med Sport. 2009; 12(1):79–84. 18068433 10.1016/j.jsams.2007.08.005

[8] Foster C, Florhaug JA, Franklin J, Gottschall L, Hrovatin LA, Parker S, Doleshal P, Dodge C. A New Approach to Monitoring Exercise Training. J Strength Cond Res. 2001; 15(1):109–15. 11708692

[9] Halouani J, Chtourou H, Gabbett T, Chaouachi A, Chamari K. Small-Sided Games in Team Sports Training: A Brief Review. J Strength Cond Res. 2014; 28(12):3594–618. 24918302 10.1519/JSC.0000000000000564

[10] Gallo T, Cormack S, Gabbett T, Williams M, Lorenzen C. Characteristics impacting on session rating of perceived exertion training load in Australian footballers. J Sports Sci. 2015; 33(5):467–75. 25113820 10.1080/02640414.2014.947311

[11] Jaspers A, De Beéck TO, Brink MS, Frencken WGP, Staes F, Davis JJ, Helsen WF. Relationships Between the External and Internal Training Load in Professional Soccer: What Can We Learn From Machine Learning? Int J Sports Physiol Perform. 2018; 13(5):625–30. 29283691 10.1123/ijspp.2017-0299

[12] Lovell TWJ, Sirotic AC, Impellizzeri FM, Coutts AJ. Factors Affecting Perception of Effort (Session Rating of Perceived Exertion) During Rugby League Training. Int J Sports Physiol Perform. 2013; 8(1):62–9. 23302138 10.1123/ijspp.8.1.62

[13] Impellizzeri FM, Rampinini E, Coutts AJ, Sassi A, Marcora SM. Use of RPE-Based Training Load in Soccer. Med Sci Sports Exerc. 2004; 36(6):1042–7. 15179175 10.1249/01.mss.0000128199.23901.2f

[14] Brink MS, Frencken WGP, Jordet G, Lemmink KAPM. Coaches’ and Players’ Perceptions of Training Dose: Not a Perfect Match. Int J Sports Physiol Perform. 2014; 9(3):497–502. 24235774 10.1123/ijspp.2013-0009

[15] Foster C, Heimann KM, Esten PL, Brice G, Porcari JP. Differences in perceptions of training by coaches and athletes. S Afri J Sports Med. 2001; 8:3–7.

[16] Bartlett JD, O’Connor F, Pitchford N, Torres-Ronda L, Robertson SJ. Relationships Between Internal and External Training Load in Team-Sport Athletes: Evidence for an Individualized Approach. Int J Sports Physiol Perform. 2017; 12(2):230–4. 27194668 10.1123/ijspp.2015-0791

[17] Geurkink Y, Vandewiele G, Lievens M, de Turck F, Ongenae F, Matthys SPJ, Boone J, Bourgois JG. Modeling the Prediction of the Session Rating of Perceived Exertion in Soccer: Unraveling the Puzzle of Predictive Indicators. Int J Sports Physiol Perform. 2019; 14(6):841–6. 30569767 10.1123/ijspp.2018-0698

[18] Marynowicz J, Lango M, Horna D, Kikut K, Andrzejewski M. Predicting ratings of perceived exertion in youth soccer using decision tree models. Biol Sport. 2022; 39(2):245–52. 35309546 10.5114/biolsport.2022.103723 PMC8919883

[19] Rossi A, Perri E, Pappalardo L, Cintia P, Iaia F. Relationship between External and Internal Workloads in Elite Soccer Players: Comparison between Rate of Perceived Exertion and Training Load. Appl Sci. 2019; 9(23):5174.

[20] Varley MC, Gabbett T, Aughey RJ. Activity profiles of professional soccer, rugby league and Australian football match play. J Sports Sci. 2014; 32(20):1858–66. 24016304 10.1080/02640414.2013.823227

[21] Brady AJ, Moyna NM, Scriney M, McCarren A. Activity profile of elite Gaelic football referees during competitive match play. Sci Med Football. 2023; 7(1):57–63. 10.1080/24733938.2022.2049456 35285413

[22] Minett GM, Fels-Camilleri V, Bon JJ, Impellizzeri FM, Borg DN. Peer Presence Increases Session Ratings of Perceived Exertion. Int J Sports Physiol Perform. 2022; 17(1):106–10. 34560668 10.1123/ijspp.2021-0080

[23] Moreira A, Bilsborough JC, Sullivan CJ, Cianciosi M, Aoki MS, Coutts AJ. Training Periodization of Professional Australian Football Players During an Entire Australian Football League Season. Int J Sports Physiol Perform. 2015; 10(5):566–71. 25405365 10.1123/ijspp.2014-0326

[24] Seiler KS, Kjerland GØ. Quantifying training intensity distribution in elite endurance athletes: is there evidence for an “optimal” distribution? Scand J Med Sci Sports. 2006; 16(1):49–56. 16430681 10.1111/j.1600-0838.2004.00418.x

[25] Hoppe MW, Baumgart C, Polglaze T, Freiwald J. Validity and reliability of GPS and LPS for measuring distances covered and sprint mechanical properties in team sports. PLoS ONE. 2018; 13(2):e0192708. 29420620 10.1371/journal.pone.0192708 PMC5805339

[26] Hopkins WG, Marshall SW, Batterham AM, Hanin J. Progressive Statistics for Studies in Sports Medicine and Exercise Science. Med Sci Sports Exerc. 2009; 41(1):3–12. 19092709 10.1249/MSS.0b013e31818cb278

[27] Weaving D, Dalton-Barron N, McLaren S, Scantlebury S, Cummins C, Roe G, Jones B, Beggs C, Abt G. The relative contribution of training intensity and duration to daily measures of training load in professional rugby league and union. J Sports Sci. 2020; 38(14):1674–81. 32314673 10.1080/02640414.2020.1754725

[28] Stellingwerff T. Case Study: Nutrition and Training Periodization in Three Elite Marathon Runners. Int J Sport Nutr Exerc Metab. 2012; 22(5):392–400. 23011657 10.1123/ijsnem.22.5.392

[29] Jamnick NA, Pettitt RW, Granata C, Pyne DB, Bishop DJ. An Examination and Critique of Current Methods to Determine Exercise Intensity. Sports Med. 2020; 50(10):1729–56. 32729096 10.1007/s40279-020-01322-8

[30] Gaudino P, Iaia FM, Strudwick AJ, Hawkins RD, Alberti G, Atkinson G, Gregson W. Factors Influencing Perception of Effort (Session Rating of Perceived Exertion) During Elite Soccer Training. Int J Sports Physiol Perform. 2015; 10(7):860–4. 25671338 10.1123/ijspp.2014-0518

[31] Marynowicz J, Kikut K, Lango M, Horna D, Andrzejewski M. Relationship Between the Session-RPE and External Measures of Training Load in Youth Soccer Training. J Strength Cond Res. 2020; 34(10):2800–4. 32773542 10.1519/JSC.0000000000003785

[32] Cullen BD, McCarren AL, Malone S. Ecological validity of self-reported wellness measures to assess pre-training and pre-competition preparedness within elite Gaelic football. Sport Sci Health. 2021; 17(1):163–72.

[33] Malone S, Hughes B, Roe M, Mangan S, Collins K. Factors that Influence Session-Rating of Perceived Exertion in Elite Gaelic Football. J Strength Cond Res. 2020; 34(4):1176–83. 32213785 10.1519/JSC.0000000000002192

[34] Daly LS, Catháin CÓ, Kelly DT. Do players with superior physiological attributes outwork their less-conditioned counterparts? A study in Gaelic football. Biol Sport. 2024; 41(1):163–74. 10.5114/biolsport.2024.129479 PMC10765432 38188097

